# Quantifying the sweetness intensity and impact of aroma in honey from four floral sources

**DOI:** 10.1111/1750-3841.17461

**Published:** 2024-10-22

**Authors:** Hannah Mulheron, Aubrey DuBois, Emily J. Mayhew

**Affiliations:** ^1^ Department of Food Science and Human Nutrition Michigan State University East Lansing Michigan USA

**Keywords:** dose‐response curves, gas chromatography‐mass spectrometry, odor‐induced taste enhancement, sucrose, sweetness intensity

## Abstract

**Abstract:**

Unlike many commercial sweeteners for which sweetness dose‐response curves have been constructed, honey's sweetness has yet to be quantified. Honey differs from most commercial sweeteners in that it has a robust aroma; this aroma may impact its perceived sweetness. This study quantified the sweetness intensity and the impact of aroma on the perceived sweetness of four different honey varieties (clover, wildflower, alfalfa, and orange) compared to sucrose. Each sweetener evaluated was diluted to six concentrations in water ranging from 12.5 g/L to 125 g/L. Panelists (*n *= 55) rated the sweetness intensities with and without aroma, in replicate, on the Global Sensory Intensity Scale. Additionally, the volatile organic compounds in the honey samples were profiled using gas chromatography‐mass spectrometry (GC/MS) analysis. Honey and sugar were equivalently sweet at a given concentration (g/L), with aroma present (*p* = 0.251). Additionally, honey and sugar were not equivalently sweet without aroma; aroma significantly increased sweetness intensities for all sweeteners (*p* = 0.042) and especially honeys. In a 100 g/L solution, the aromas in honey increased its sweetness by 23%–43%, depending on the floral source. Compounds with sweet aroma characteristics were identified at high concentrations in all honey samples using GC/MS analysis, including furfural, benzaldehyde, benzene acetaldehyde, and dimethyl sulfide. Additionally, (S)‐limonene and toluene were present in high quantities in the orange and alfalfa samples. This study can inform appropriate honey usage levels and identify major volatiles that may enhance sweetness.

**Practical Application:**

Honey sweetness has not been determined quantitatively, despite the widespread use of honey among consumers and product formulators. Sweetness enhancement by honey aroma volatiles may support a reduction in added sugars while maintaining sweetness intensity.

## 1 INTRODUCTION

Honey is a sweetener with a long history of use, but its sweetness intensity has yet to be quantified. Honey is derived from plant nectar collected by honeybees and is comprised of sugar, water, protein, enzymes, organic acids, vitamins, minerals, pigments, and phenolic compounds. The exact ratio of sugars, as well as the other constituents of honey, varies by the botanical source, climate, processing, and more (Da Silva et al., 2016). Approximately 80% of honey's composition consists of sugars, and of those, approximately 75% are monosaccharides (Da Escuredo et al., 2013; Kozłowicz et al., 2020; Silva et al., 2016). In addition to monosaccharides, researchers have identified more than 25 disaccharides and oligosaccharides in various honeys, such as sucrose, palatinose, trehalose, and melibiose (de la Fuente et al., 2007; Kaškoniene & Venskutonis, 2010; Sanz et al., 2004).

Most simple carbohydrates bind to the Venus Flytrap (VFT) sites of the T1R2 and T1R3 taste receptors, triggering the delivery of neurotransmitters to the afferent cranial nerve, which the brain processes as tasting sweet (Fernstrom et al., 2012). Although the processing mechanism is the same, different carbohydrates elicit different sweetness intensities. Some sweeteners can reach a greater maximum intensity, while others are more potent. For example, fructose and sucrose have a similar maximum sweetness intensity, and both sweeteners elicit a much stronger sweetness intensity than glucose (Clemens et al., 2016). On the other hand, aspartame is much more potent than sucrose, and significantly less aspartame is needed than sucrose to achieve a similar sweetness intensity (Wee et al., 2018). The construction of a dose–response curve, which models sweetness intensity as a function of sweetener concentration, is a common way to represent the sweetness intensity of a sweetener. A known relationship between concentration and sweetness intensity is crucial for food product producers, consumers, and nutritionists to target a specific sweetness intensity in foods and meals (Wee et al., 2018).

An additional factor that impacts sweetness intensity is aroma. A growing body of research has found that certain aromas can influence the perception and intensity of basic tastes (sweet, salt, sour, bitter, and umami) (Bartoshuk & Klee, 2013; Frank & Byram, 1988; Spence, 2022; Wang et al., 2018, 2019; Zhang et al., 2023). Eating and drinking is a multimodal sensory experience that combines gustatory, olfactory, and somatosensory sensory systems (Small, 2012). The cognitive experience of flavor combines inputs from taste and retronasal olfaction (odors emitted from foods in the mouth), and there is often an overlap in the reception of these sensory inputs (Bartoshuk & Klee, 2013; Prescott, 1999; Small, 2012). Many of the aromas that have been reported as sweet taste enhancers are typically paired with sweet taste, such as fruity and floral aromas (Bartoshuk & Klee, 2013; Frank & Byram, 1988; Spence, 2022). Vanillin, the primary odor compound in vanilla, is a hallmark example of an aroma compound with this effect (Ventura & Mennella, 2011; Wang et al., 2018; Yeomans et al., 2008). Using sweet‐enhancing volatiles is a promising strategy for reducing added sugars in food (Hopfer et al., 2022).

Unlike most other commercial sweeteners, honey has an intrinsic aroma. Its aroma is commonly described as sweet, floral, citrus, medicinal, and woody (Siegmund et al., 2018). The aroma composition of honey varies by floral source, and researchers have identified over 600 volatile organic molecules in various kinds of honey (Jerković et al., 2009). The most common chemical classes of volatiles identified in honey include aldehydes, ketones, acids, alcohols, esters, hydrocarbons, and sulfurous compounds (Jerković et al., 2009; Kaškoniene & Venskutonis, 2010). Aldehydes that are commonly identified in honey include benzaldehyde (characteristic of almonds), phenylacetaldehyde (sweet, rose, green, and grassy), heptanal (fatty and pungent odor), hexenal (strong green grass), nonanal (orange, fatty, and aldehydic), and furfural (sweet and woody) (Jerković et al., 2009; Piasenzotto et al., 2003; Radovic et al., 2001; Siegmund et al., 2018; Silva et al., 2016). Terpenes such as linalool and its derivatives like lilac alcohol and lilac aldehyde have also been identified in many honeys (Jerković et al., 2009; Piasenzotto et al., 2003). Individual compounds have been identified as markers for particular honey varieties, such as methyl anthranilate, a marker of Spanish citrus honey (Piasenzotto et al., 2003; Soria et al., 2003; White & Bryant, 1996).

So, how sweet is honey, and how do its aromas impact its sweetness? In this study, we aim to characterize the sweetness of honey in three parts. First, we quantified the relative sweetness of honey from four floral sources: alfalfa, wildflower, orange, and clover. To our knowledge, this study represents the first construction of sweetness dose‐response curves for honeys. Second, we quantified the impact of total aroma on honey sweetness. Third, we identified key volatile organic compounds present in the honey samples that may impact sweetness.

## 2 MATERIALS AND METHODS

### 2.1 Quantification of honey sweetness and the impact of aroma

#### 2.1.1 Sweet taste solutions

Sugar (Domino pure cane sugar) and four varieties of honey (clover, alfalfa, wildflower, and orange; Dutch Gold, Lancaster Co.) were each diluted to six concentrations in reverse osmosis purified water (Besco Water Treatment, Inc.) (hereafter referred to as “concentration set”). Each concentration set consisted of 12.5, 25, 50, 75, 100, and 125 g of sweetener/L. Concentration set doses are also reported in terms of volume (tsp/cup), calories (kcal/cup), and comparable consumer beverages in Table S1. Solutions were prepared in 1 L batches, pumped into 2 oz black cups (15 mL servings), and labeled with random three‐digit codes. Prepared samples were kept refrigerated and brought to room temperature for 30 min before serving. Honey samples were stored in 1 lb bottles and kept frozen (−20°C) until sample preparation; honey was not used beyond 7 days post thawing.

#### 2.1.2 Sensory test design

Human subjects (*n* = 66) were recruited to participate in a training/screening session to qualify for six sample evaluation sessions to rate sweetness intensities on the Global Sensory Intensity Scale (GSIS) (Hudson et al., 2018). Training on the GSIS took place in small groups and began by asking panelists to think of their strongest remembered sensation of any kind. This sensation was used as their personal top anchor for the scale (numerically 100). Next, they were asked to rate the intensities of 18 remembered sensations, such as “the brightness of a dimly lit room” and “strongest oral pain experienced” and place a dash and numerical value on a hard copy of the scale for each sensation; remembered sensations used for training were adapted from Bartoshuk et al. (2002). Panelists shared their ratings in a facilitated group discussion and were encouraged to adjust their scale usage as needed. Next, they rated the intensities of 12 physical stimuli. Stimuli included various tastes, trigeminal sensations, and sounds; six of the physical stimuli were rated for sweet taste intensity (carrot, Ritz cracker (Mondelez International), banana, Coke (The Coca‐Cola Company), marshmallow (Kraft Heinz), and 100 g/L sweet taste solution. Finally, they transferred their ratings onto a digital scale, administered through the RedJade sensory software (RedJade Sensory Solutions LLC). Individuals were screened for understanding based on correct rankings of select remembered sensations and physical stimuli (e.g., a carrot is less sweet than Coke and Coke is less sweet than remembered “strongest sweetness experienced”). Of the 66 individuals who completed the training, 55 met the criteria and completed the full study design. 

The remainder of the study consisted of six sessions, during which panelists (*n* = 55) rated the sweetness intensity of solutions; surveys were administered via RedJade. Panelists evaluated 20 concentration sets, rating each sweetener type four times. Panelists evaluated samples in sessions 1–3 without intervention (“with aroma”), while in sessions 4–6, panelists wore nose clips (Frienda, purchased via Amazon.com) to block olfactory perception (“without aroma”). At the beginning of each session, panelists completed a warm‐up exercise in which they rated the intensity of three basic taste solutions, namely: sweet (100 g sugar/1 L H_2_O), sour (1 g citric acid/1 L H_2_O), and salty (3.5 g NaCl/1 L H_2_O). Panelists rated 3–4 concentration sets of solutions per session (three sets in sessions 1, 2, 4, and 5; four sets in sessions 3 and 6). To minimize sources of variation and potential biases, the presentation order of concentration sets and samples within a set was randomized via a complete block design; however, each set always began with the 50 g/L concentration of the set. Additionally, sampling protocol was standardized: panelists were instructed to sip the entire 15‐mL sample, swish in their mouth for 5 s, expectorate the sample, and then give the intensity rating, with an enforced 30‐s break between samples and 3 min between sets. During that time, they rinsed their mouth with room temperature water and expectorated into a spit cup.

Panelists were compensated with a $15 e‐gift card for participating in the training session and a $10 e‐gift card/session for the remainder of the study. The Michigan State University IRB approved all sensory testing protocols for this study (STUDY00007723), and all participants gave informed consent prior to enrollment in the study.

#### 2.1.3 Data analysis

All analyses were performed using R version 4.3.2 (2023‐10‐31 ucrt). Dose‐response curves relating sweetness intensity to sweetener concentration were fit using a Hill equation using the drc package (v3.0.1; Ritz et al., 2015) for sugar and all four honey varieties, and curves were visualized using ggplot2 (v3.4.4; Wickham, 2016). Dose‐response curves were used to extract the relative sweetness of honey and iso‐sweet concentrations of honey compared to sucrose. We conducted a mixed‐effect model analysis of variance (ANOVA) to analyze the effect of aroma, sweetener, concentration, and subject and their 2‐way and 3‐way interactions on sweetness intensity. We conducted a second mixed‐effect model ANOVA excluding sweetness intensity ratings without aroma, analyzing the effect of sweetener, concentration, and subject, and their 2‐way and 3‐way interactions (afex v1.3‐1). To determine in which cases aroma significantly enhanced sweetness, we conducted paired *t*‐tests on sweetness intensity ratings with and without aroma for individual sweeteners at each concentration tested. ANOVAs and *t*‐tests were performed using the stats package (v4.3.2; R Core Team 2023), and a significance level of *α* = 0.05 was used for all comparisons.

### 2.2 Determination of volatile organic compounds in honey

#### 2.2.1 Sample preparation and GC/MS protocol

The volatile profiles of each honey were characterized using headspace‐solid phase microextraction (HS‐SPME) gas‐chromatography/mass‐spectrometry (GC/MS). Four samples were prepared for each honey variety; two commercial honey bottles for each variety were analyzed, with two technical replicates prepared from each bottle. To prepare the honey samples for extraction, 8 g of honey along with equal parts of a saturated NaCl solution were combined in 40 mL vials equipped with a mininert valve (Supelco Inc.), and heated in a 40°C hot water bath for 10 min. Following heating, vials were vortexed for 10 s and equilibrated at room temperature for 5 min. Volatiles were extracted from the headspace of prepared samples for 10 min using a manual SPME device (Supelco Inc.) equipped with a 65 µm polydimethylsiloxane/divinylbenzene fiber (Supelco Inc.). Following extraction, the SPME fiber was inserted into the splitless inlet (200°C) of the gas chromatograph (Agilent 6890 Hewlett‐Packard Co.), coupled to a mass spectrometer (Pegasus III TOF MS with Agilent 6890 GC, LECO) and desorbed for 30 s (Park et al., 2024). During desorption, the first 20 cm of the column (HP‐5 60 m × 0.25 mm) were cyrofocused using liquid nitrogen (Song et al., 1998). The oven program began at 40°C and increased to 280°C at a rate of 43°C/min, for a total run time of 7 min. The column carrier gas, helium, had a constant flow rate of 1.50 mL/min. Additionally, the SPME fiber was kept in the injector port for the entirety of the run to desorb all volatiles from the fiber and ensure there was no carryover of volatiles between samples.

#### 2.2.2 Volatile standards

A standard blend was prepared with 0.4 µL each of the following 19 volatiles: acetone, 1‐hexanol, phenylethyl alcohol, benzyl alcohol, decanal, furan, furfural, heptane, linalool, myrcene, octanal, o‐xylene, phenylacetaldehyde, S‐(‐)‐limonene (Sigma Aldrich), hexanal, benzaldehyde (Aldrich Chemical Co.), 2‐methyl butanal (Alfa Aesar), dimethyl disulfide (Tokyo Kasei), and nonanal (Fluka Chemika Corp.). Two‐tenths of a microliter of the standard blend was added to a 20 mm filter paper (Gelman Instrument Co.) and dropped into a custom gas‐tight 4.4 L volumetric flask with a Mininert valve (Alltech Assoc., Inc.); the blend was incubated in the flask for 24 h to allow complete vaporization of the standards (Song et al., 1997).

Chromatographs were analyzed using LECO deconvolution software (Song et al., 1997). The identification of volatile compounds was determined by comparing retention times of external standard compounds and mass fragmentation spectra patterns from the National Institute for Standard and Technology spectral library (version 2.0) for all compounds reported. Only compounds with a high probability match (>5000) in at least two replicates or compounds identified using an external standard are reported.

## 3 RESULTS AND DISCUSSION

### 3.1 Quantification of honey's relative sweetness

**FIGURE 1:**
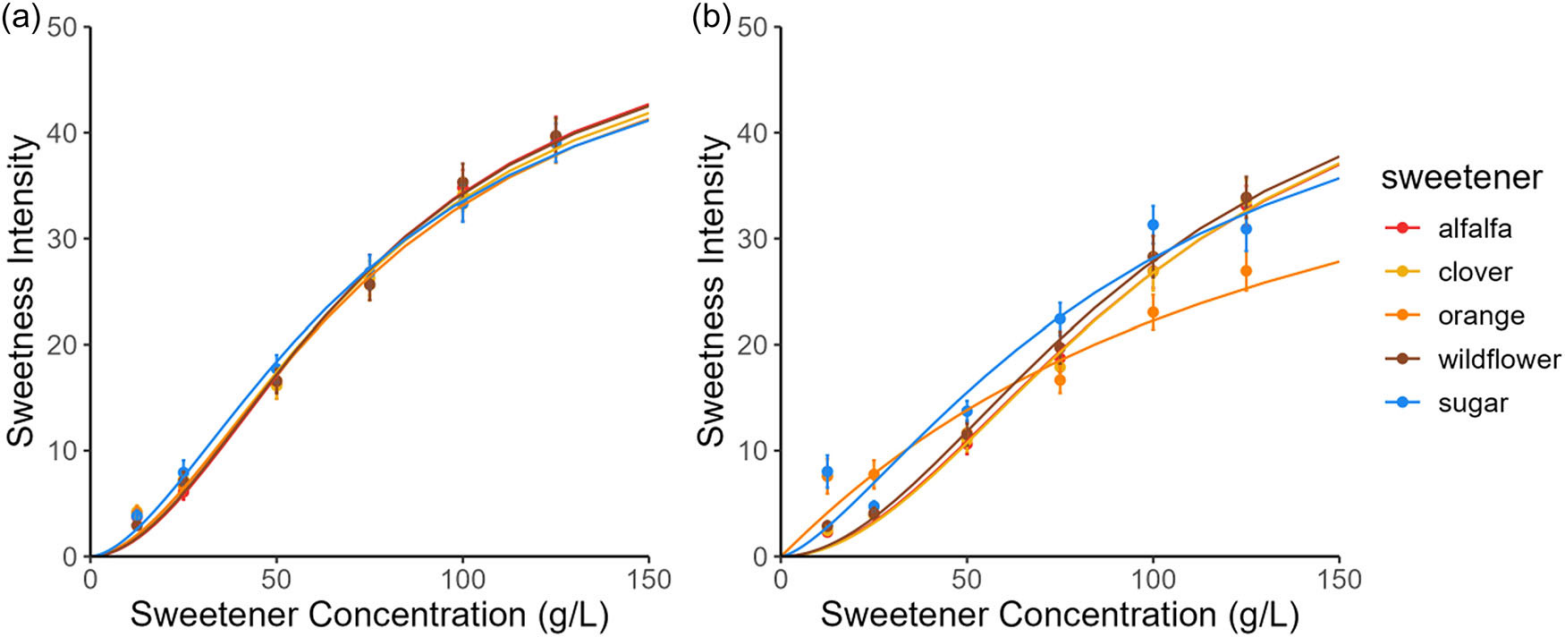
Mean sweetness intensity ratings (a) with aroma and (b) without aroma as a function of sweetener concentration by mass (g/L) for all sweeteners (alfalfa honey, clover honey, orange honey, wildflower honey, and sugar). Error bars represent the standard error of the mean (±1 SE).

The average sweetness intensity ratings for each sweetener with aroma are visually represented as sweetness dose‐response curves (Figure 1). There was no statistical difference in average sweetness intensity ratings between all five sweeteners rated with aroma (Figure 1; Table S2). The coefficients of the fitted Hill equation used to define the dose‐response curves for each sweetener are shown in Table 1. When the maximum response is not constrained, the generated maximum values are unrealistic for sweetness intensities when using a generalized scale (up to 88, with 100 representing the strongest sensation of any kind). Consequently, we constrained the maximum to 53.6, the average rating among our subject pool for “strongest sweetness ever experienced.” The sweetness intensity of each sweetener measured, with aroma, can be estimated at any concentration within the experimental range using the dose‐response curve equation (Table 1).

**TABLE 1:** Hill equation and its coefficients for sweetness intensity dose‐response curves of solutions rated with aroma. *X* represents the concentration of a sweetener (g/L), *n* represents the Hill coefficient, and EC_50_ is the concentration (g/L) that produces a 50% maximum response. Maximum response was constrained to 53.6.

$$\begin{array}{c} f\left(x\right)=\frac{1}{{\left(1+\frac{EC_{50}}{x}\right)}^{n}} \end{array}$$		
**Sweetener**	**EC_50_ (g/L)**	***n***
Alfalfa honey	79.2	1.77
Clover honey	75.9	1.81
Orange honey	77.3	1.78
Wildflower honey	75.3	1.89
Sugar	74.6	1.67

Knowing the potency of a sweetener is essential for recipe development and food formulation. In addition, it is common for consumers to measure sweetener content in terms of volume (tsp added to foods at home) or nutritional profile (kcal and g of added sugars in packaged foods). Notably, honey is approximately 70% more dense than sugar (Table S3), meaning 1 tsp of honey contains more sugar than 1 tsp of table sugar. The sugar content of the honey samples used in this study ranged between 79 and 81 g sugars/100 g honey, with the majority coming from the monosaccharides glucose (32%–37%) and fructose (38%–41%) (Table S4) (Oroian, 2013; Zhu et al., 2024). On the other hand, ordinary table sugar is at least 99% sucrose, a polysaccharide containing one glucose and one fructose unit. One factor that may affect the differences in sweetness intensity between the sweeteners is the composition of saccharides, as sucrose is less sweet than fructose per unit mass (Wee et al., 2018). In addition to differences in sugar content, honey has a reported caloric density that is 21% lower than that of sucrose (sugar: 3.85 kcal/g; honey 3.04 kcal/g) (FoodData Central, 2019b, 2019a). Since honey and sugar are equivalently sweet per unit mass, 21% fewer calories are needed to achieve the same sweetness when using honey (Table S5).

The implications of the differences in volumetric and caloric densities between the two sweeteners are shown in Table 2. Given the 1.7x higher density of honey, the same sweetness as 1 tsp (or 1 Tbsp or cup) of sugar can be achieved with approximately 40% lower volume of honey. Table 2 can be used as a guide for consumers, dietitians, and product developers who are looking to substitute sugar with honey. However, these equivalencies are based on the sweeteners alone in water and do not account for the functional role of sucrose, loss of aroma volatiles through cooking and baking, and interactions with other aromas and tastants from foods. Dose‐response curves that express sweetness intensity as a function of volumetric sweetener concentration (mL/L) and caloric density (kcal/L) are provided (Figure S2–S3).

**TABLE 2:** Equivalently sweet volumes of honey and sugar; grams of total sugars and kcal of reported volumes.

Equivalently sweet concentrations		Grams of sugars			kcal		
Sugar	Honey	Sugar	Honey	Difference	Sugar	Honey	Difference
1 tsp	0.58 tsp	4.19	3.34	0.85	16.2	12.8	3.4
1 Tbsp	0.58 Tbsp	11.98	9.53	2.45	46.2	36.48	9.72
1 cup	0.58 cup	199	158	41	770	608	162

*Note*: Values were calculated using the average densities (1.43 g/mL, 0.83 g/mL), sugar content (0.794 g/g, 0.998 g/g), USDA kcal values (3.04 kcal/1 g, 3.85 kcal/1 g) for honey and sugar, respectively. Our measured densities differ slightly from USDA reported density.

### 3.2 Quantification of the impact of aroma volatiles on honey's relative sweetness

**FIGURE 2:**
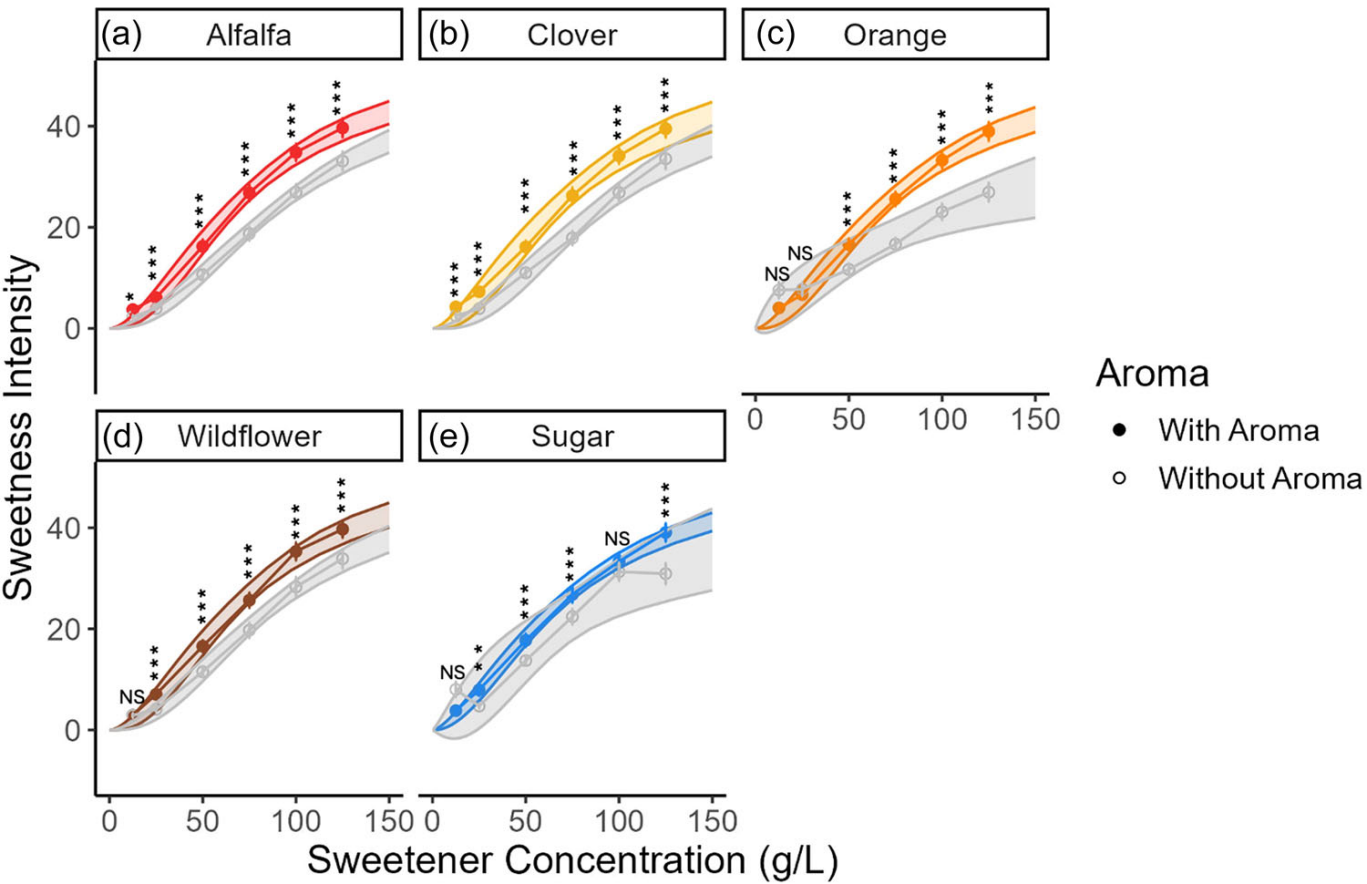
Mean sweetness intensity ratings as a function of sweetener concentration (g/L) with and without aroma for each of the sweeteners: (a) alfalfa honey, (b) clover honey, (c) orange honey, (d) wildflower honey, and (e) sugar. Error bars represent the standard error of the mean (±1 SE). Ratings with aroma that are statistically greater than ratings without aroma are defined as ^NS^ *p* ≥ 0.05, *p < 0.05, **p < 0.01, and ***p < 0.001.

The differences in saccharide composition between the sweeteners may be responsible for honey and sugar eliciting equivalent sweetness intensities despite a 20% difference in sugar content; however, taste‐aroma interactions are an essential factor to consider. Evaluation of changes in sweetness intensity ratings given with and without nose clips provides further evidence for the impact of aroma on perceived sweetness (Figures 1b and 2). Aroma significantly affected perceived sweetness across sweeteners (*p* = 0.042).

At 100 g/L, comparable to the sugar concentration of Coke (Table S1), the aromas in alfalfa, clover, orange, and wildflower honey enhanced sweetness by 27.9%, 25.5%, 42.6%, and 23.1%, respectively; in comparison, the aromas in sugar enhanced sweetness by 5.4% (Table S6). The decrease in sweetness ratings for sugar without aroma, which has a very faint aroma (Urbanus et al., 2014), could be due to an overall muting of sensory input when the nasal cavity and aroma perception were blocked. Without aroma, orange honey was the sweetest at low concentrations but the least sweet at higher concentrations. One potential explanation for this result may be the presence of other tastants, such as acids, that suppress sweetness perception (Spence, 2022). This finding also suggests that the impact of aroma on the sweetness of honey is highly variety‐specific, and our results may not translate to all varieties of honey. Honeys with robust tastant profiles in particular may deviate from our observed trends; for example, we may see lower sweetness intensity for a honey variety like buckwheat, which contains more than double the amount of phenolic compounds, which are bitter tastants, than the tested honeys (Oroian, 2013; Zhu et al., 2024).

All the honey varieties are less sweet than sugar without aroma but are equivalently sweet with aroma; this observation indicates that taste‐aroma interactions significantly impact perceived sweetness and suggests that the volatiles present in honey enhance sweetness. Note that 100 g of honey has 20.6 g of sugar and 81 kcal <100 g of sugar, yet the two sweeteners are equivalently sweet; the aromas in honey are bridging the gap in sugar disparities. Although substituting sugar for honey may present a strategy for reducing sugar without sacrificing sweetness that can be implemented both by consumers and the food industry, it may not be suitable for all food and beverage applications, either because the flavor of honey does not fit the desired flavor profile or due to functional differences between the sweeteners. Further testing utilizing honey in food and beverage mediums needs to be conducted to characterize the effect of food system on the potency of sweetness enhancement from honey aroma.

### 3.3 Determination of volatile organic compounds in honey

GC/MS results reveal that the orange honey had the most diverse aroma profile (87 compounds identified), while the clover had the fewest distinct aroma compounds profile (64 compounds identified); a complete list of compounds identified in each honey variety is provided in Tables S7–S10. The 15 most abundant volatile organic compounds (VOCs) found in the headspace of any of the tested honey varieties, along with any additional VOCs confirmed using an external standard, are shown in Table 3. Furfural, benzaldehyde, dimethyl sulfide, and phenylacetaldehyde comprised a large percentage of the total ion count (TIC) in each of the four honey varieties, and all these compounds have a sweet aroma characteristic. Other sweet‐smelling compounds identified at lower concentrations in each variety include 3‐methyl‐2‐butenal, decanal, and phenylethyl alcohol. Many other aroma characteristics were congruent with sweetness (e.g., caramel, fruity, cherry, and apple). Other commonly identified aroma families that may or may not be congruent with sweetness include floral, fatty, vegetable, and musty.

**TABLE 3:** Average %total ion chromatogram (TIC) of the 15 most abundant compounds in any of the tested honey varieties (alfalfa, orange, clover, and wildflower) and any additional compounds that were identified using an external standard.

Compound name	Alfalfa (%TIC)	Orange (%TIC)	Clover (%TIC)	Wildflower (%TIC)	Aroma characteristic
(s)‐Limonene^a^	10.26	21.11	–	–	Camphoraceous; herbal; terpenic
Furfural^a^	4.49	5.23	8.86	8.40	Sweet; caramellic; bready
Benzaldehyde^a^	5.98	4.51	8.37	4.98	Sweet; cherry; maraschino cherry
Toluene	2.80	8.04	–	–	Sweet
Dimethyl sulfide	1.16	1.36	6.50	4.92	sweet; vegetable; sulfurous
Phenylacetaldehyde^a^	0.94	1.47	5.74	3.90	sweet; fermented; floral
Pivaloyl acetonitrile	4.67	–	1.31	–	–
Ethanol	2.08	2.88	0.36	1.61	alcoholic; medicinal; ethereal
Dimethyl disulfide^a^	0.52	0.81	3.99	1.47	vegetable; onion; cabbage
3‐Penten‐2‐ol	4.13	0.07	1.97	0.52	vinyl; green
3‐Methylbutanal	1.05	0.96	1.77	2.57	aldehydic; fatty; ethereal
(+)‐Neoisomenthol	1.72	1.19	1.61	–	mentholic; musty; woody
2‐Butenal, 2‐methyl‐,(E)‐	2.60	0.06	1.63	–	–
2‐Methylbutanal^a^	0.67	0.54	1.82	2.23	malty; musty; fermented
cis‐Linalool oxide	0.68	3.94	0.18	0.36	floral
Dimethyl silanediol	1.12	0.53	1.45	1.33	–
P‐Menth‐1‐en‐9‐al	–	1.08	–	–	herbal; spicy
Octane	0.97	0.86	1.49	0.82	–
Acetic acid	–	0.33	–	1.46	sour; acidic; vinegar
Hotrienol	0.43	2.34	0.29	0.47	tropical
Nonanal^a^	0.78	1.09	0.71	0.87	aldehydic; fatty; cucumber
3‐Hepten‐2‐one	0.19	1.22	–	–	–
3‐Methyl‐3‐buten‐1‐ol	1.24	–	0.63	0.15	sweet; fermented; yeasty
Acetone^a^	0.51	0.34	0.60	0.99	apple; solvent; pear
3‐Methyl‐2‐butenal	1.25	0.02	0.73	0.15	sweet; cherry; nutty
Decamethylcyclopentasiloxane	0.21	0.07	1.17	0.17	–
Myrcene^a^	0.08	0.63	–	–	spicy; peppery; plastic
2‐Butanol	–	0.03	–	0.60	sweet; fruity; apricot
Decanal^a^	0.20	0.29	0.22	0.43	sweet; aldehydic; floral
2‐Butanone	0.19	–	0.14	0.46	camphoraceous; acetone; fruity
Octanal^a^	0.30	0.19	0.22	0.33	aldehydic; fatty; herbal
Benzyl alcohol^a^	–	–	0.32	0.18	sweet; floral; fruity
Hexanal^a^	0.25	–	–	0.25	vegetable; aldehydic; clean
Phenylethyl alcohol^a^	0.48	0.06	0.31	0.14	sweet; dried rose; floral
Heptane^a^	0.10	–	0.15	–	sweet; ethereal
O‐Xylene^a^	0.07	0.05	0.03	0.10	geranium
Furan^a^	0.04	0.04	0.05	0.03	ethereal
1‐Hexanol^a^	0.04	–	–	–	sweet; pungent; herbal

^a^ Identification confirmed with an external standard.

(S)‐limonene is the most abundant compound in the orange and alfalfa honey (21.11% and 10.26% TIC), and toluene is also present at high levels in both samples (8.04% and 2.80% TIC); however, neither VOC was identified in clover or wildflower honey. Interestingly, (s)‐limonene has a “camphoraceous, herbal, and terpenic” aroma, which is not necessarily congruent with sweetness. The aromas in the orange honey contributed to a 40%, or greater, increase in sweetness intensity at a concentration of 50 g/L and beyond. Toluene has a sweet aroma characteristic and is the second most abundant compound in the orange honey. This poses the question of whether the concentration of an aroma compound, its aroma characteristic, the complexity of the mixture, or the combined aroma character is a more significant factor contributing to sweet taste enhancement by aroma volatiles. Further work exploring sweetness enhancement with individual compounds present at high and low concentrations (e.g., (s)‐limonene and phenylethyl alcohol) will help broaden the understanding of the sweetness‐enhancing ability of honey's aromas.

## 4 CONCLUSION

When measured in units of mass, sugar and all four honey varieties were iso‐sweet in water across the concentration range measured with aroma present, representing the full range of typical commercial sweetened beverages. However, honey is a more potent sweetener per unit of energy, delivering an equivalent sweetness intensity with 21% fewer kcals. Consumers must use a lower volume of honey when substituting it for sugar to reap these nutritional benefits since it is 70% more dense than sugar. On a volumetric (tsp) basis, consumers can use 42% less honey to achieve similar sweetness (i.e., 0.58 tsp of honey per 1 tsp of granulated table sugar).

We found that aromas meaningfully contributed to the total sweetness intensity of honey. Sugar is sweeter than honey when olfactory perception is blocked. However, this difference disappears when honey aroma is present—the aromas in honey bridge the gap in the perceived intensity of the tastants. Generally, honey or aromatic sweeteners could be a valuable nutritional strategy to reduce added sugar intake without sacrificing the sweetness of foods. However, this study only characterized aqueous solutions, and these results do not account for more complex interactions in food systems.

Through GC‐MS analysis, ≥64 aroma compounds were identified in the headspace of each honey variety. (S)‐limonene, furfural, and benzaldehyde were the most abundant volatiles in the tested honey samples. Knowing that honey aromas enhance sweetness, future studies should investigate the contribution of specific honey volatiles to sweetness intensity. If the sweetness‐enhancing effect of honey aroma can be attributed to finite set of aroma volatiles, these compounds can be strategically used in food formulations.

## AUTHOR CONTRIBUTIONS

**Hannah Mulheron**: Methodology; formal analysis; writing—original draft; investigation; data curation; visualization. **Aubrey DuBois**: Methodology; formal analysis; writing—review and editing; investigation; data curation. **Emily J. Mayhew**: Conceptualization; methodology; supervision; formal analysis; writing—review and editing; funding acquisition; data curation; visualization.

## CONFLICT OF INTEREST STATEMENT

The authors declare no conflicts of interest.

## Supporting information



Supplemental Materials
