# Supplementary material for: Quantifying the sweetness intensity and impact of aroma in honey from four floral sources
Source: J Food Sci. 2024 Oct 22;89(12):9732–41. doi: 10.1111/1750-3841.17461 (PMC11673473; doi:10.1111/1750-3841.17461)
Supplement: Supplementary file 1 — Supplemental Materials [file JFDS-89-9732-s001.docx]

**Supplemental Materials**

**Table S1:** Concentrations of experimental concentration set by volume (tsp/cup), calories (kcal/cup), and comparable consumer products.

| **Experimental concentrations (g/L) ​** | **Concentration by Volume ​** | | **Concentration by Calories ​** | | **Comparable consumer product ​** |
| --- | --- | --- | --- | --- | --- |
|  | **Sucrose**​ **(tsp/cup)**​ | **Honey**​**(tsp/cup)**​ | **Sucrose**​ **(kcal/cup)**​ | **Honey**​ **(kcal/cup)**​ |  |
| 12.5​ | 0.70​ | 0.41​ | 11.38​ | 8.98​ | Pure Leaf Subtly Sweet Iced Tea (9g/L)​ |
| 25​ | 1.40​ | 0.81​ | 22.75​ | 17.97​ | C20 Non-GMO Coconut Water The Original (29g/L)​ |
| 50​ | 2.79​ | 1.62​ | 45.51​ | 35.93​ | Lipton Iced Tea with Lemon (50g/L)​ |
| 75​ | 4.21​ | 2.43​ | 68.26​ | 53.90​ | IZZE Clementine Sparkling Juice (77g/L)​ |
| 100​ | 5.60​ | 3.24​ | 91.02​ | 71.87​ | Coke (106g/L)​ |
| 125​ | 7.00​ | 4.07​ | 113.77​ | 89.83​ | Simply Lemonade (117g/L)​ |

**Table S2:** Mixed-effects model ANOVA results showing the effect of subjects, sweetener concentration (g/L), and their 2-way and 3-way interactions for sweetness ratings with aroma.

|  | Df | F-value | p-value |
| --- | --- | --- | --- |
| Subject | 54 | 91.4 | ~0.00* |
| Concentration | 5 | 231 | 2.7e-95 |
| Sweetener | 4 | 0.25 | 0.91 |
| Subject:Concentration | 270 | 7.20 | 3.1e-148 |
| Subject:Sweetener | 216 | 1.67 | 3.5e-8 |
| Concentration:Sweetener | 20 | 1.19 | 0.85 |
| Subject:Concentration:Sweetener | 1080 | 0.57 | 0.25 |

**p-value generated in R is 0.00 because it is lower than the minimum value computed in R, exact p-value is unknown.*

**Table S3.** Measured density in g/mL for each of the five sweeteners (mean ± standard error).

| **Sweetener** | **Sugar** | **Average of all 4 honeys** | **Alfalfa Blossom** | **Wildflower Blossom** | **Orange Blossom** | **Clover** |
| --- | --- | --- | --- | --- | --- | --- |
| **Density (g/mL)** | 0.83 ± 0.038 | 1.43± 0.013 | 1.42 ± 0.140 | 1.42 ± 0.159 | 1.43 ± 0.126 | 1.44 ± 0.084 |

**Table S4:** Average fructose, glucose, sucrose, and total carbohydrate content ± standard deviation of three samples tested in triplicate. The data presented are summarized from Zhu et al. (2024) Table S2h for Alfalfa1, Clover3, Wildflower2, and Orange1.

|  | Alfalfa | Clover | Orange | Wildflower |
| --- | --- | --- | --- | --- |
| Fructose | 36.60 ± 1.28 | 40.36 ± 1.89 | 38.25 ± 0.41 | 41.35 ± 1.90 |
| Glucose | 32.49 ± 0.90 | 32.03 ± 0.95 | 31.78 ± 0.34 | 36.98 ± 0.82 |
| Sucrose | 0.42 ± 0.04 | 0.55 ± 0.08 | 0.68 ± 0.02 | 0.11 ± 0.01 |
| Total carbohydrate content | 79.30 ± 5.55 | 78.52 ± 2.28 | 78.34 ± 1.97 | 80.67 ± 1.66 |

**Table S5**: Volume and caloric comparison of equivalently sweet sugar and honey doses. Honey and table sugar are equivalently sweet by mass. The honeys used in this study have an average sugar content of 0.794g/g; the density of honey is 1.72 times that of sugar (1.43g/mL vs 0.83g/mL); the caloric density of sugar is 1.27 times that of honey (3.85kcal/g vs 3.04kcal/g).

| Equivalently sweet concentrations​ | | Table sugar (sucrose) ​ | | Honey​ | | |
| --- | --- | --- | --- | --- | --- | --- |
| Sugar​ (tsp/ 8 oz. cup)​ | **Honey (tsp/ 8 oz. cup)**​ | **Grams of sugars**​ | **Kcal**​ | **Grams**​ | **Grams of sugars**​ | **Kcal**​ |
| 1​ | 0.58 | 4.2​ | 16.2​ | 4.2​ | 3.3 | 12.8 |
| 2​ | 1.16 | 8.4​ | 32.4​ | 8.4​ | 6.7 | 25.5​ |
| 3​ | 1.74 | 12.6​ | 48.5​ | 12.6​ | 10.0 | 38.3 |

**
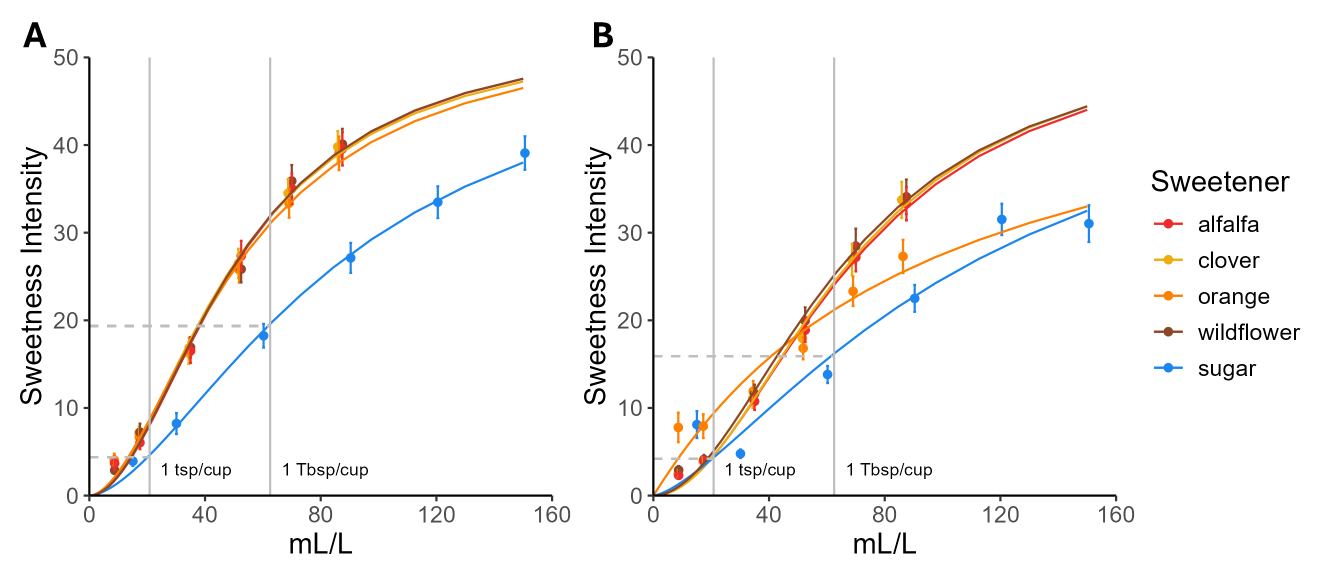
**

**Figure S2:** Mean sweetness intensity ratings **(A)** with aroma and **(B)** without aroma as a function of sweetener concentration by Volume (mL/L) (vertical lines represent the concentrations that are equivalent to 1 tsp/8 oz. cup and 1 Tbsp/8 oz. cup of each sweetener/ cup of water) for all 5 sweeteners (Alfalfa Honey, Clover Honey, Orange Honey, Sucrose, and Wildflower Honey). Error bars represent the standard error of the mean (±1 se).


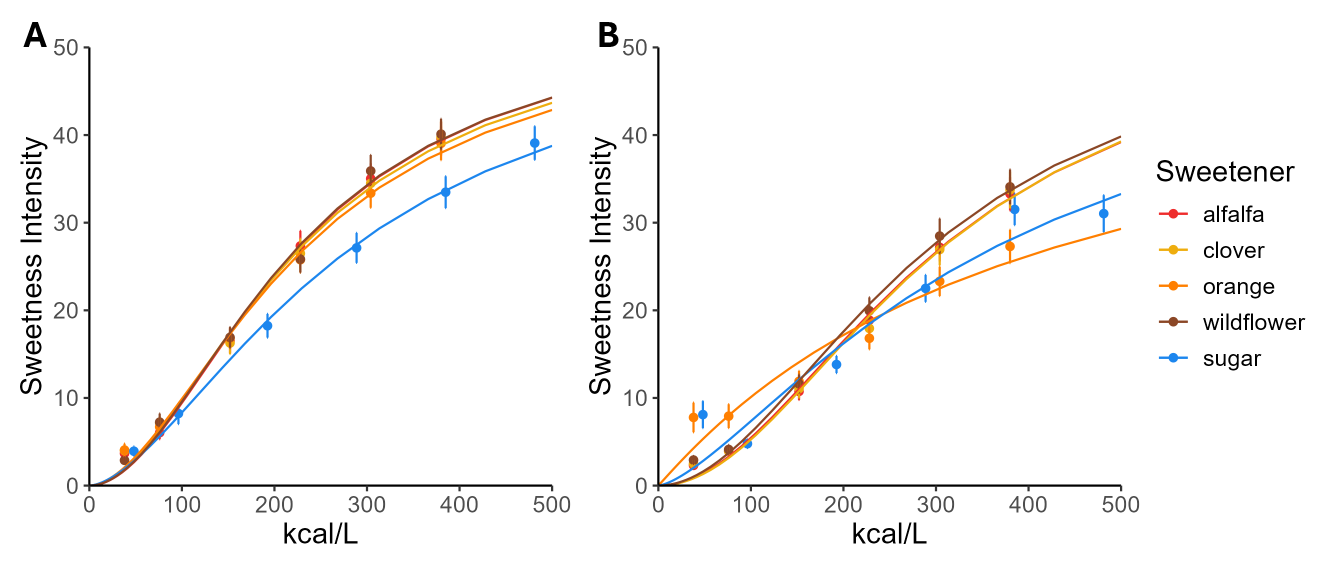


**Figure S3:** Mean sweetness intensity ratings **(A)** with aroma and **(B)** without aroma as a function of sweetener concentration by Calories (kcal/L) for all 5 sweeteners (Alfalfa Honey, Clover Honey, Orange Honey, Sucrose, and Wildflower Honey). Error bars represent the standard error of the mean (±1 se).

**Table S6:** Percent increase in sweetness intensity at each concentration set level for all 5 sweeteners (Alfalfa Honey, Clover Honey, Orange Honey, Sucrose, and Wildflower Honey).

| Sweetener Concentration (g/L) | Sweetener | | | | |
| --- | --- | --- | --- | --- | --- |
|  | **Alfalfa** | **Clover** | **Orange** | **Sucrose** | **Wildflower** |
| 12.5 | 60.6 | 68.6 | -47.4 | -53.0 | 0.07 |
| 25 | 53.1 | 82.3 | -14.9 | 65.3 | 70.2 |
| 50 | 50.3 | 44.6 | 40.3 | 27.3 | 41.7 |
| 75 | 40.9 | 44.7 | 51.3 | 18.1 | 28.5 |
| 100 | 27.9 | 25.5 | 42.6 | 5.4 | 23.1 |
| 125 | 18.6 | 16.3 | 43.4 | 25.1 | 15.8 |

**Table S7:** Percent total ion count (TIC) of compounds identified in the headspace of alfalfa honey.

| No. | Compound name | avg %TIC |
| --- | --- | --- |
| 1 | Acetaldehyde | 0.06 |
| 2 | Ethanol | 2.08 |
| 3 | Trimethylborane | 0.02 |
| 4 | Acetone*†* | 0.51 |
| 5 | Furan*†* | 0.04 |
| 6 | 1,4-Pentadiene | 0.38 |
| 7 | Dimethyl sulfide | 1.16 |
| 8 | 2-methyl propanal | 0.31 |
| 9 | 2,3-Butanedione | 0.77 |
| 10 | 2-Butanone | 0.19 |
| 11 | 3-Penten-2-ol | 4.13 |
| 12 | Trichloromethane | 0.11 |
| 13 | Isobutyronitrile | 0.82 |
| 14 | 2-methyl-2-Propen-1-ol | 0.06 |
| 15 | 3-methylbutanal | 1.05 |
| 16 | 1-Butanol | 0.03 |
| 17 | 2-methylbutanal*†* | 0.67 |
| 18 | 1-hydroxy-2-propanone | 0.07 |
| 19 | Dimethyl silanediol | 1.12 |
| 20 | 2,3-Pentanedione | 0.13 |
| 21 | Pentanal | 0.08 |
| 22 | Heptane*†* | 0.10 |
| 23 | Bromodichloromethane | 0.16 |
| 24 | 3-hydroxy-2-butanone | 0.34 |
| 25 | 2-methylbutanenitrile | 0.68 |
| 26 | 3-methyl-3-buten-1-ol | 1.24 |
| 27 | 3-methyl- 1-butanol | 0.69 |
| 28 | Hydroxymethyl 2-hydroxy-2-methylpropionate | 0.02 |
| 29 | 2,5-dimethylfuran | 0.01 |
| 30 | (E)-2-methyl-2-butenal | 2.60 |
| 31 | Dimethyl disulfide*†* | 0.52 |
| 32 | Methyl 1-methylcyclopropyl ketone | 0.14 |
| 33 | Methallyl cyanide | 0.05 |
| 34 | 3-methylpentanal | 0.15 |
| 35 | Toluene | 2.80 |
| 36 | Butanoic acid | 0.16 |
| 37 | 3-methyl-2-butenal | 1.25 |
| 38 | 3-Hepten-2-one | 0.19 |
| 39 | Octane | 0.97 |
| 40 | Hexanal*†* | 0.25 |
| 41 | Dibromochloromethane | 0.08 |
| 42 | Hexamethylcyclotrisiloxane | 0.16 |
| 43 | Furfural*†* | 4.49 |
| 44 | 4,4-Dimethyl-3-oxopentanenitrile | 4.67 |
| 45 | (Z)-3-Hexen-1-ol | 0.55 |
| 46 | 1-Hexanol*†* | 0.04 |
| 47 | Ethylbenzene | 0.14 |
| 48 | o-Xylene*†* | 0.07 |
| 49 | Oxime-, methoxy-phenyl-_ | 0.04 |
| 50 | 2-Cyclopentene-1,4-dione | 0.03 |
| 51 | 2-Acetylfuran | 0.32 |
| 52 | Heptanal | 0.08 |
| 53 | 2-Heptanone | 0.14 |
| 54 | Benzocyclobutene | 0.04 |
| 55 | 5-methylfurfural | 0.05 |
| 56 | Benzaldehyde*†* | 5.98 |
| 57 | 1-Octen-3-ol | 0.15 |
| 58 | Dimethyl trisulfide | 0.07 |
| 59 | Octamethylcyclotetrasiloxane | 0.04 |
| 60 | Myrcene*†* | 0.08 |
| 61 | Octanal*†* | 0.30 |
| 62 | (S)-Limonene*†* | 10.26 |
| 63 | Phenylacetaldehyde*†* | 0.94 |
| 64 | cis-Linalool oxide | 0.68 |
| 65 | trans- Linalool oxide | 0.24 |
| 66 | Nonanal*†* | 0.78 |
| 67 | Hotrienol | 0.43 |
| 68 | Phenylethyl Alcohol*†* | 0.48 |
| 69 | Lilac aldehyde** | 0.02 |
| 70 | Decamethylcyclopentasiloxane | 0.21 |
| 71 | Benzyl nitrile | 0.05 |
| 72 | (+)-neoisomenthol | 1.72 |
| 73 | Decanal*†* | 0.20 |
| 74 | Methyl Salicylate | 0.09 |
| 75 | 3-Phenylfuran | 0.03 |
| 76 | Damascenone | 0.03 |

*Spectral match to NIST,* *†* *identification confirmed with external standard, ** correct isomer not identified.*

**Table S8:** Percent total ion count (TIC) of compounds identified in the headspace of clover honey.

| No. | Compound Name | avg %TIC |
| --- | --- | --- |
| 1 | Acetaldehyde | 0.03 |
| 2 | Methanethiol | 0.03 |
| 3 | Ethanol | 0.36 |
| 4 | Acetone*†* | 0.60 |
| 5 | Furan*†* | 0.05 |
| 6 | Dimethyl Sulfide | 6.50 |
| 7 | 2-methylpropanal | 0.28 |
| 8 | 2,3-Butanedione | 0.41 |
| 9 | 2-Butanone | 0.14 |
| 10 | 3-Penten-2-ol | 1.97 |
| 11 | Trichloromethane | 0.10 |
| 12 | Isobutyronitrile | 0.27 |
| 13 | 3-methylbutanal | 1.77 |
| 14 | 2-methylbutanal*†* | 1.82 |
| 15 | 1-hydroxy-2-propanone | 0.06 |
| 16 | Dimethyl silanediol | 1.45 |
| 17 | 2,3-Pentanedione | 0.10 |
| 18 | Heptane*†* | 0.15 |
| 19 | Bromodichloromethane | 0.16 |
| 20 | 2-methylbutanenitrile | 0.38 |
| 21 | 3-Buten-1-ol, 3-methyl- | 0.63 |
| 22 | 3-methyl-1-butanol | 0.51 |
| 23 | 1-Butanol, 2-methyl-, (S) | 0.11 |
| 24 | 2-Butenal, 2-methyl-,(E)- | 1.63 |
| 25 | Dimethyl disulfide*†* | 3.99 |
| 26 | Methallyl cyanide | 0.05 |
| 27 | 3-methylpentanal | 0.12 |
| 28 | Spiro[2.4]hepta-4,6-diene | 0.17 |
| 29 | 3-Penten-2-ol | 0.66 |
| 30 | Butanoic acid | 0.67 |
| 31 | 3-methyl-2-butenal | 0.73 |
| 32 | Octane | 1.49 |
| 33 | Dibromochloromethane | 0.08 |
| 34 | Hexamethyl cyclotrisiloxane | 0.14 |
| 35 | 3-Methylbutanoic acid | 0.15 |
| 36 | Furfural*†* | 8.86 |
| 37 | 4,4-Dimethyl-3-oxopentanenitrile | 1.31 |
| 38 | (z)-3-Hexen-1-ol | 0.31 |
| 39 | o-xylene*†* | 0.03 |
| 40 | Oxime-, methoxy-phenyl-_ | 0.05 |
| 41 | 2-Cyclopentene-1,4-dione | 0.03 |
| 42 | Nonane | 0.50 |
| 43 | Heptanal | 0.04 |
| 44 | 2-Acetylfuran | 0.28 |
| 45 | 5-methylfurfural | 0.05 |
| 46 | Benzaldehyde*†* | 8.37 |
| 47 | 1-Octen-3-ol | 0.24 |
| 48 | Dimethyl trisulfide | 0.74 |
| 49 | Octamethylcyclotetrasiloxane | 0.04 |
| 50 | Octanal*†* | 0.22 |
| 51 | Benzyl Alcohol | 0.32 |
| 52 | Phenylacetaldehyde*†* | 5.74 |
| 53 | Dihydromyrcenol | 0.15 |
| 54 | trans-Linalool oxide | 0.18 |
| 55 | 1-methyl-4-(1-methylethenyl)-benzene | 0.71 |
| 56 | Nonanal*†* | 0.71 |
| 57 | Hotrienol | 0.29 |
| 58 | Phenylethyl Alcohol*†* | 0.31 |
| 59 | Decamethylcyclopentasiloxane | 1.17 |
| 60 | (+)-neoisomenthol | 1.61 |
| 61 | Decanal*†* | 0.22 |
| 62 | Methyl Salicylate | 0.03 |
| 63 | 3-phenyl furan | 0.03 |
| 64 | Damascenone | 0.03 |

*Spectral match to NIST,* *†* *identification confirmed with external standard, ** correct isomer not identified*

**Table S9:** Percent total ion count (TIC) of compounds identified in the headspace of orange honey.

| No. | Compound Name | avg %TIC |
| --- | --- | --- |
| 1 | Acetaldehyde | 0.04 |
| 2 | Ethanol | 2.88 |
| 3 | Trimethylborane | 0.07 |
| 4 | Acetone*†* | 0.34 |
| 5 | Furan*†* | 0.04 |
| 6 | Dimethyl sulfide | 1.36 |
| 7 | Methane sulfonyl chloride | 0.01 |
| 8 | 2-methylpropanal | 0.18 |
| 9 | Formic acid | 0.03 |
| 10 | 2,3-Butanedione | 0.30 |
| 11 | 3-Methyl-2-pentanone | 0.11 |
| 12 | 2-Butanol | 0.03 |
| 13 | Acetic acid | 0.33 |
| 14 | Trichloromethane | 0.04 |
| 15 | Isobutyronitrile | 0.32 |
| 16 | 2-Methyl-1-propanol | 0.06 |
| 17 | 3-Penten-2-ol | 0.07 |
| 18 | 3-methylbutanal | 0.96 |
| 19 | 1-Butanol | 0.04 |
| 20 | 2-methylbutanal*†* | 0.54 |
| 21 | 1-hydroxy-2-propanone | 0.03 |
| 22 | 2,3-Pentanedione | 0.10 |
| 23 | Dimethyl silanediol | 0.53 |
| 24 | Pentanal | 0.02 |
| 25 | Bromodichloromethane | 0.07 |
| 26 | 3-Hydroxy-2-butanone | 0.14 |
| 27 | 2-Methylbutanenitrile | 0.23 |
| 28 | 3-Methylbutanenitrile | 1.04 |
| 29 | 2-Methyl-1-butanol | 0.16 |
| 30 | 2-Methyl-2-butenal | 0.06 |
| 31 | Dimethyl disulfide*†* | 0.81 |
| 32 | 2-Methyl-3-pentanone | 0.03 |
| 33 | 3-Methylpentanal | 0.02 |
| 34 | Toluene | 8.04 |
| 35 | 3-methyl-2-butenal | 0.02 |
| 36 | 3-Hepten-2-one | 1.22 |
| 37 | Octane | 0.86 |
| 38 | 2-Ethylcyclobutanol | 0.34 |
| 39 | Dibromochloromethane | 0.04 |
| 40 | 3(2H)-Furanone, dihydro-2-methyl- | 0.10 |
| 41 | Hexamethyl cyclotrisiloxane | 0.09 |
| 42 | 3-Methyl-butanoic acid | 0.04 |
| 43 | Acetyl valeryl | 0.07 |
| 44 | Furfural*†* | 5.23 |
| 45 | Ethylbenzene | 0.37 |
| 46 | o-Xylene*†* | 0.05 |
| 47 | Oxime-, methoxy-phenyl-_ | 0.04 |
| 48 | 2-Methylpentanoic acid | 0.04 |
| 49 | Cyclopropane, propyl- | 0.06 |
| 50 | 3-Hydroxy-2-butanone | 0.01 |
| 51 | 2-Furanmethanol | 0.01 |
| 52 | Heptanal | 0.09 |
| 53 | 2-Acetylfuran | 0.34 |
| 54 | 2-methyl-3-Octyne- | 0.11 |
| 55 | Pentanoic acid, 2-hydroxy-4-methyl-, methyl ester | 0.06 |
| 56 | 2-Ethylhexanal | 0.01 |
| 57 | Lilac alcohol** | 0.02 |
| 58 | 5-Methylfurfural | 0.15 |
| 59 | Benzaldehyde*†* | 4.51 |
| 60 | 1-Octen-3-ol | 0.07 |
| 61 | Dimethyl trisulfide | 0.03 |
| 62 | Octamethylcyclotetrasiloxane | 0.03 |
| 63 | Myrcene*†* | 0.63 |
| 64 | Octanal*†* | 0.19 |
| 65 | (S)-Limonene*†* | 21.11 |
| 66 | Phenylacetaldehyde*†* | 1.47 |
| 67 | Cis-Linalool oxide | 3.94 |
| 68 | Nonanal*†* | 1.09 |
| 69 | Hotrienol | 2.34 |
| 70 | Phenylethyl Alcohol*†* | 0.06 |
| 71 | Decamethylcyclopentasiloxane | 0.07 |
| 72 | Benzyl nitrile | 0.17 |
| 73 | Lilac aldehyde** | 0.39 |
| 74 | 2,6,6-Trimethyl-2-cyclohexene-1,4-dione | 0.06 |
| 75 | 2H-Pyran-3-ol, 6-ethenyltetrahydro-2,2,6-trimethyl- | 0.17 |
| 76 | (+)-neoisomenthol | 1.19 |
| 77 | Terpinen-4-ol | 0.01 |
| 78 | Anethofuran | 0.27 |
| 79 | P-Menth-1-en-8-ol | 0.32 |
| 80 | Decanal*†* | 0.29 |
| 81 | 2-Thiophenecarboxylic acid, 3-methyl-, methyl ester | 0.02 |
| 82 | Safranal | 0.07 |
| 83 | P-Menth-1-en-9-al | 1.08 |
| 84 | 3-Phenylfuran | 0.01 |
| 85 | Nonanoic acid | 0.07 |
| 86 | Damascenone | 0.04 |

*Spectral match to NIST,* *†* *identification confirmed with external standard, ** correct isomer not identified*

**Table S10:** Percent total ion count (TIC) of compounds identified in the headspace of wildflower honey.

| No. | Compound Name | avg %TIC |
| --- | --- | --- |
| 1 | Acetaldehyde | 0.07 |
| 2 | Methanethiol | 0.03 |
| 3 | Ethanol | 1.61 |
| 4 | Acetone*†* | 0.99 |
| 5 | Furan*†* | 0.03 |
| 6 | Dimethyl sulfide | 4.92 |
| 7 | 2-Methylpropanal | 0.45 |
| 8 | Formic acid | 0.12 |
| 9 | 2,3-Butanedione | 0.53 |
| 10 | 2-Butanone | 0.46 |
| 11 | (R)-2-Butanol | 0.60 |
| 12 | 3-Penten-2-ol | 0.52 |
| 13 | Acetic acid | 1.46 |
| 14 | Trichloromethane | 0.13 |
| 15 | 2-Methyl-1-propanol | 0.21 |
| 16 | Isobutyronitrile | 0.04 |
| 17 | 3-Methylbutanal | 2.57 |
| 18 | 2-Methylbutanal*†* | 2.23 |
| 19 | 1-Hydroxy-2-propanone | 0.04 |
| 20 | 2,3-Pentanedione | 0.06 |
| 21 | Dimethyl silanediol | 1.33 |
| 22 | 2,5-Dimethylfuran | 0.01 |
| 23 | Bromodichloromethane | 0.20 |
| 24 | 3-Hydroxy-2-butanone | 0.20 |
| 25 | 2-Methylbutanenitrile | 0.22 |
| 26 | 3-Methyl-3-buten-1-ol | 0.15 |
| 27 | 3-Methyl-1-butanol | 0.50 |
| 28 | Dimethyl disulfide*†* | 1.47 |
| 29 | 2-Methyl-1-butanol | 0.29 |
| 30 | Methallyl cyanide | 0.07 |
| 31 | 3-Methyl-pentanal | 0.05 |
| 32 | Butanoic acid | 0.07 |
| 33 | 3-Methyl-2-butenal | 0.15 |
| 34 | Octane | 0.82 |
| 35 | Hexanal*†* | 0.25 |
| 36 | Dibromochloromethane | 0.10 |
| 37 | 3(2H)-Furanone, dihydro-2-methyl- | 0.07 |
| 38 | Hexamethylcyclotrisiloxane | 0.17 |
| 39 | 3-Methylbutanoic acid | 0.10 |
| 40 | Furfural*†* | 8.40 |
| 41 | (Z)-3-Hexen-1-ol | 0.32 |
| 42 | Hexanenitrile | 0.13 |
| 43 | Oxime-, methoxy-phenyl-_ | 0.06 |
| 44 | o-Xylene*†* | 0.10 |
| 45 | Heptanal | 0.11 |
| 46 | 2-Acetylfuran | 0.45 |
| 47 | 2-Ethylhexanal | 0.02 |
| 48 | 5-Methylfurfural | 0.09 |
| 49 | Benzaldehyde*†* | 4.98 |
| 50 | 1-Octen-3-ol | 0.27 |
| 51 | Dimethyl trisulfide | 0.08 |
| 52 | Octamethylcyclotetrasiloxane | 0.05 |
| 53 | Octanal*†* | 0.33 |
| 54 | Benzyl Alcohol | 0.18 |
| 55 | Phenylacetaldehyde*†* | 3.90 |
| 56 | Dihydromyrcenol | 0.17 |
| 57 | cis-Linalool oxide | 0.36 |
| 58 | Nonanal*†* | 0.87 |
| 59 | Hotrienol | 0.47 |
| 60 | Phenylethyl Alcohol*†* | 0.14 |
| 61 | Isophorone | 0.15 |
| 62 | Decamethylcyclopentasiloxane | 0.17 |
| 63 | Lilac aldehyde** | 0.01 |
| 64 | Octanoic Acid | 0.18 |
| 65 | Ethanone, 1-(1,4-dimethyl-3-cyclohexen-1-yl)- | 0.07 |
| 66 | Decanal*†* | 0.43 |
| 67 | Methyl salicylate | 0.02 |
| 68 | Myrtenal | 0.07 |
| 69 | 3-phenylfuran | 0.02 |
| 70 | Nonanoic acid | 0.12 |
| 71 | Damascenone | 0.04 |

*Spectral match to NIST,* *†* *identification confirmed with external standard, ** correct isomer not identified*
